# Sustainability in Orthodontics: what can we do to save our planet?

**DOI:** 10.1590/2177-6709.22.4.113-117.sar

**Published:** 2017

**Authors:** Matheus Melo Pithon, Lucianne Cople Maia de Faria, Orlando Motohiro Tanaka, Antônio Carlos de Oliveira Ruellas, Laura Salignac de Souza Guimarães Primo

**Affiliations:** 1 Universidade Federal do Rio de Janeiro, Programa de Pós-graduação em Odontopediatria e Ortodontia (Rio de Janeiro/RJ, Brasil).; 2 Pontifícia Universidade Católica do Paraná, Programa de Pós-graduação em Odontologia com ênfase em Ortodontia e Ortopedia Facial (Curitiba/PR, Brasil).

**Keywords:** Sustainability, Environment, Orthodontics.

## Abstract

The sustainability of the natural resources of our planet is a topic for worldwide debate. Mankind, during its evolution as a species, has not been greatly concerned about conserving the environment in which we live. Nowadays we are reaping the fruits of this neglect. Climatic changes and storms are good examples of this. We, humans, must re-think our attitudes in order to leave the planet in a healthy state to be used by our descendants. But thinking of orthodontics, what can we do as orthodontists? From this perspective, the authors of the present study aimed, in a clear and objective manner, to present simple and sustainable ways to proceed during our activity as orthodontists, in order to minimize the effects on nature, caused by man.

## RESUMO

A sustentabilidade dos recursos naturais de nosso planeta é tema de debate em todo o mundo. O homem, durante a evolução como espécie, pouco se preocupou em conservar o meio ambiente em que vive. Nos dias de hoje, colhemos os frutos do descaso; mudanças climáticas e tempestades são bons exemplos disso. Nós, seres humanos, devemos repensar nossas atitudes, a fim de deixar o planeta propício para que nossos descendentes possam utilizá-lo. Mas, pensando-se na Ortodontia, o que nós ortodontistas podemos fazer? Nessa perspectiva, os autores do presente estudo se propuseram a apresentar, de forma clara e objetiva, formas simples e sustentáveis para serem utilizadas durante nossa atividade como ortodontistas, a fim de minimizar os efeitos causados pelo homem na natureza.

## INTRODUCTION

There is eminent need for greater concern and care with regard to the management of human resources and environmental impacts arising from human activities.^1^ ^,^ ^2^ From November 30 through December 11, 2015, several Heads of State of diverse nationalities met in France, during the 21st United Nations Climate Change Conference, (COP21) to discuss strategies for minimizing global warming, CO_2_ emission and the use of environmental resources.

The relationship between health and ecology has, over the last few years, become an important topic to researchers in quest of measures for the preservation of life on the planet.^3^ ^,^ ^4^ In this scenario, dental practice not only interacts in oral health-disease, but generates solid health residues that include heavy metals and biomedical waste potentially harmful to the environment, leading to growing concern about the management of these residues.^5^ ^,^ ^6^ Therefore, arising from these factors is sustainable dentistry, which implements sustainable practices for maintaining the level of consumption of resources in harmony with the economy of nature, diminishing the environmental impacts by reducing or eliminating the dejecta and chemical products released into the environment.^3^ ^,^ ^7^

The four processes responsible for the largest portion of waste and pollution from dental practice are: use of materials containing mercury (amalgam fragments and waste - mercury vapor release); conventional X-ray systems (equipment, film, silver fixer, insoluble developer solutions); infection control methods including disposable barriers, toxic products for sterilization and disinfectants; conventional vacuum saliva suction systems.^8^ ^,^ ^9^ Thus, green dentistry is based on the model of four-Rs: rethink, reduce, reuse and recycle.^10^ Thus, the authors’ proposal in this article is to clearly and objectively present strategies that we, as orthodontists, could implement to make our clinical practice more sustainable, and consequently reduce devastation of the planet.

### What should we do?

The first step towards practicing sustainable dentistry consists of rethinking consciousness and attitudes; changing the way in which the dental office is seen; implementing simple changes by taking into consideration that this is a continuous process. In addition, the team must be trained, with each member doing his/her part and knowing the sustainable practices.^7^ ^,^ ^11^ This includes the manner in which the dental office is planned or when it is being refurbished. At this time, the plan should seek: to make maximum use of natural light; opt for materials with organic constituents; equipment and lighting with the lowest electrical power consumption (fluorescent or LED lamps). Another good option would be the use of solar energy that in addition to being a clean source of electrical power, will reduce the value of the bill.

Thus, sustainable practice in the dental office begins right from the time patients enter the reception, until the time of their clinical attendance. Therefore, the reception room must undergo changes for improved sustainable practice, thus diminishing the volume of trash generated by disposable materials. Other practices such as cleaning the air conditioner filters; use of a rain water collection system; use of motivational stickers against wasting water; use of a toilet basin that saves water. These are important items for performing eco-friendly dentistry. 

Table 1 illustrates how some habits are carried out in the reception room; how sustainable practice must be performed, and the benefit this brings to the environment. In the restrooms of the reception as well as in the reserved to the professionals, sustainable practice must also be inserted as demonstrated in Table 2. In the dental office itself, the use of lighting components is responsible for high electrical power consumption, therefore, when thinking about sustainable alternatives we are not only reducing the energy expended, but we are also saving money. 

**Table 1:** The benefits arising from a sustainable practice in the dental office reception room

How it is done	Sustainable practice	Benefit
Use of disposable cups	Use of corn starch or glass cups	Less production of solid residues
Use of incandescent or fluorescent lamps	Use of LED lamps^12^	Lower electrical power consumption up to 80%
Use of switches	Use of movement sensors in less frequently used areas.	Lower electrical power consumption
Use conventional paper for printing	Use recyclable paper	Making best use of resources
Furniture made of synthetic, non-recyclable materials	Use of furniture made of reforested wood	Lower emission of gases into the atmosphere, and these are biodegradable
Printing in normal mode	Use draft mode	50% saving of ink
Magazines and newspapers on paper or plastic	Tables with access to Internet so patients can entertain themselves before being attended to	Elimination of solid residues
Throw paper into the garbage can together with other types of materials	Throw paper away in selective trash collectors	Reduction of solid residues and possibility of recycling
Use of electronic appliances without power consumption classification	Use of electronic appliances with low energy consumption	Lower electrical power consumption
Artificial plants and ornamentation	Use real plants	Avoids the use of plastic materials and promotes transformation of CO_2_ into O_2_ through photosynthesis by plants
Keep the computer switched on all day	Switch off the monitor when not in use	Electricity saving
Use of tube, LCD or plasma television sets	Use LED technology television sets	Lower electrical power consumption with savings of up to 80%

**Table 2:** The benefits arising from a sustainable practice in the rest rooms of the dental office.

How it is done	Sustainable practice	Benefit
Use of toilet paper	Use of hygienic shower with recyclable toilet paper	Less production of solid residues
Use of incandescent or fluorescent lamps	Use of LED lamps^12^	Lower electrical power consumption up to 80%
Use of conventional faucets with threaded spindle to close them.	Use faucets with automatic closing system	Water saving
Paper towel	Electric dryer for hands	Less production of solid residues
Conventional detergents	Biodegradable detergents	Lower quantity of toxic residues in water

Moreover, there is a great use for uncontaminated solid residues, such as the use of disposable materials and sterilization items. Inadequate management at the time of discarding them is responsible for exacerbated trash production; therefore, it is essential to implement sustainable practices to diminish the quantity of trash produced, or for better re-use of these materials. Table 3 illustrates sustainable practices related to the sterilization process.

**Table 3:** The benefits arising from a sustainable practice during the process of sterilization of dental materials.

What is done	Sustainable practice	Benefit
Use of chemical materials in disinfection	Use of sterilization by steam	Reduction of toxic garbage
Use of paper bibs on patients	Use of sterilizable cloth bibs	Reduction in solid residues
Sterilize only one or a few materials in an auto-clave cycle	Sterilize several materials together in one autoclave cycle	Electrical power and water saving
Use plastic bags for packing the materials to be autoclaved	Use FDA-registered reusable pouches and wraps for sterilization	Saves money and reduces solid residues

If water and energy consumption is not properly managed it will be responsible for eminent risks to the environment. Table 4 illustrates how sustainable practices in the dental office's consulting room may replace habits that are harmful to the planet, and what benefits come from it.

**Table 4:** The benefits arising from a sustainable practice in the dental office consulting room.

What is done	What can be changed	What is saved
Use of mechanical chair	Use of automatic chair with pre-programmed commands	Shorter time of movement, that is, less energy spent
Halogen light reflector	LED light reflector	Energy saving to the order of 35%
Use of disposable suction devices throughout the procedure	Use of paper cups, as this material is biodegradable, or use of suction devices made of paper	Energy saving with regard to use of the compressor for performing suction, and lower quantity of residues
Use of conventional panoramic and periapical radiographs	Use of digital radiographs	Savings of water, energy and reduction of solid residues arising from the process of development and storage of radiographs
Prefer disposable instruments and materials	Prefer reusable and sterilizable materials and instruments	Lower amount of garbage
Patients records on paper, stored in plastic folders	Digital file of all patients’ documentation	Eliminates the use of paper and plastic, as well as economy of storage space
Faucet with stop-cock for opening	Faucet with action on pedal	Saves water and reduces cross contamination

### Sustainable orthodontics

In orthodontic practice, it is also necessary to adopt means that reduce aggression to the environment. The materials used in orthodontics must be re-thought and chosen based on the environmental impact they may cause. Use of self-etching adhesive systems, for example, dispenses the washing step required when conventional adhesive systems are used; thus reducing water consumption in this procedure. This is only one example of what we could do during our attendance. Table 5 presents other ways of performing orthodontic treatment in a sustainable manner, bringing benefits to our planet without affecting the efficacy of orthodontic treatment.

**Table 5:** How it is done, what must be done, and the benefits arising from a sustainable orthodontic practice.

What is done	Sustainable practice	Benefit
Orthodontic accessories sold in conventional packaging	Brackets sold in receptacles with a larger quantity of accessories, with these receptacles being manufactured of a recyclable product	Elimination of packaging made of plastic materials, being replaced with biodegradable materials (Fig 1).
Adhesive systems with acid etching	Self-etching adhesive systems^13^	Lower water consumption due to no need for washing and drying, with same clinical efficacy
Conventional brackets	Self-ligating brackets^14^	Less chair time and eliminates the use of elastomers
Use of non-sterilizable orthodontic archwires.	Use of orthodontic archwires capable of being sterilized^15^	Reduce discard of solid residues that may have been contaminated before use in the patient
Rebond new brackets when they debond during treatment	Recycle brackets by roughening their base with aluminum oxide and performing new bonding^16-18^	Eliminate solid residues that would go to the trash can, making it possible for them to have a longer useful life
Light polymerization with conventional halogen or LED appliances	Ultra-rapid LED light polymerizers^19^	Shorter chair time and use of LED lamp with low energy consumption
Use of synthetic intermaxillary elastics	Use of elastics made of latex^20^	Latex is extracted from a tree, consequently there is need to cultivate trees, therefore, the more widespread the use of latex, the larger the number of trees
The use of a new mini-implant in a patient who needs to replace the one in use	Sterilization and use of the same mini-implant that was removed in the same patient.^21^	Reduction of solid residues that are constituents of the mini-implant

New sustainable practices can and must be added to the list described here, so that the quest for sustainability will be a constant among all of us in the practice of the profession.

**Figure 1:**
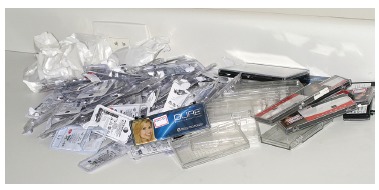
Orthodontic material packaging that will become garbage.

## CONCLUSION

Thus, it can be perceived that from individual and collective consciousness of the team, the practice of sustainable orthodontics, protecting the environment, is possible; saving money as well as the environment, helping in recovering the planet by reducing the environmental impacts generated by its practice, here including the care taken with the use of natural resources. 
